# Identifying Individuals at Risk for Fracture in Guatemala

**DOI:** 10.1371/journal.pone.0028042

**Published:** 2011-11-29

**Authors:** Keaton M. Nasser, Alejandro Quiñónez Obiols, Stuart L. Silverman

**Affiliations:** 1 Osteoporosis Medical Center, Beverly Hills, California, United States of America; 2 Universidad Mariano Gálvez de Guatemala, Guatemala City, Guatemala; 3 Cedars-Sinai/UCLA, Los Angeles, California, United States of America; University Medical Center Rotterdam, The Netherlands

## Abstract

**Introduction:**

The FRAX calculator combines a set of clinical risk factors with country-specific incidence rates to determine the ten-year absolute risk of major osteoporotic fracture. However, regional or country-specific databases from Central American countries are not available. We compared the use of various FRAX databases and the Pluijm algorithm in determining risk of fracture.

**Methods:**

We collected clinical risk factor data needed for the FRAX calculator and Pluijm algorithm of Hispanic women in Guatemala and calculated the FRAX absolute risk measures of major osteoporotic fracture and hip fracture. Subjects were postmenopausal women greater than age 40 with no history of using medication that affect bone. A random sample of 204 women in 34 different regions women in Guatemala City was visited in their homes to complete the surveys. The Pluijm risk score and FRAX risk score using the US Hispanic, Spain, and Mexican databases were calculated.

**Results:**

We used the US NOF guidelines for treatment which suggest a treatment threshold for patients with a 10-year hip fracture probability ≥3% or a 10-year major osteoporotic fracture risk ≥20%. The number of patients meeting the suggested threshold limits for treatment using the Spain and Mexico calculators were identical. There was 100% conformity in threshold limits for both hip and major osteoporotic fracture risk. The mean conformity for any fracture risk between US Hispanic and the other two databases was 97.5%. Conformity was 99.0% based on major osteoporotic fracture and 97.5% based on risk of hip fracture. The Pluijm evaluation shows conformity of 87.2% and 83.3%, respectively, when compared to the US Hispanic and Spain/Mexico FRAX thresholds for risk of fracture.

**Discussion:**

Although the different FRAX databases provide variations in the absolute risk of fracture, the overall conformity to treatment thresholds amongst the US Hispanic, Spain, and Mexico databases show the database used would have little effect as to the decision to treat. The Pluijm tool conforms to the FRAX thresholds and can be used as well. It does not matter which country-specific calculator or assessment tool is used, as there are a similar number of patients that would meet the intervention threshold.

## Introduction

Clinical risk factors are associated with increased probability for osteoporotic related fractures. A number of algorithms utilizing clinical risk factor data exist to estimate the absolute risk of osteoporotic fracture. The WHO Task Force, Garvan Institue, and other researchers have identified a superset of clinical risk factors that can be used alone or in combination with BMD results to predict the absolute risk of hip fracture and/or clinical fracture [1]. These risk factors have been combined with country-specific incidence rates into the web-based Fracture Risk Assessment Tool (FRAX) [2] to determine the ten year absolute risk of major osteoporotic (hip, clinical vertebral, forearm, and humerus) and hip fracture. The FRAX calculator has multiple databases, including several European countries and ethnic databases for the US. According to a recently issued article, Guatemalan post menopausal urban women (over 50 years) have a 27.94% risk of hip fracture and a 23.28% risk of general osteoporotic fracture at 10-year determined by FRAX using Hispanic US population database [3]. However, more regional or country-specific databases from Central American countries are necessary to confirm the utility of these instruments. We collected risk factor data from women in Guatemala City to compare the interchangeability of the various internet-based FRAX algorithms and compared them to the simpler manual algorithm developed by Pluijm et al. [4].

## Materials and Methods

Ethics Statement: This research protocol was approved by the Western Institutional Review Board (WIRB). Participants provided verbal consent. Verbal consent was permitted by the WIRB as this study was conducted via a simple questionnaire that had no chance of effect or harm on any research subject. A completed and returned questionnaire represents acceptance of consent. The clinical investigation adhered to the principles expressed in the Declaration of Helsinki.

We collected clinical risk factor data (Table 1) needed for the FRAX calculator and Pluijm algorithm of Hispanic women in Guatemala and calculated the FRAX absolute risk measures of major osteoporotic fracture and hip fracture. Subjects were postmenopausal women greater than age 40 with no history of using medication that affect bone. A random sample of 204 women in 34 different regions women in Guatemala City was visited in their homes to complete the surveys. Due to literacy, a member of the study staff completed the survey with the participants in an oral interview. Only subjects identified as Hispanic white were included in calculations to decrease heterogeneity.

**Table 1:** Characteristics of study subjects.

	mean
Age (yrs)	60±12
Height (cm)	152±7.3
Weight (kg)	62.5±12.4
BMI	27±4.6

Risk Factor	# (%)n = 204
Fragility fracture	55 (27)
Parental hip fracture	15 (7.4)
alcohol	2 (1)
smoker	7 (3.4)
Glucocorticoid use	10 (4.9)
Rheumatoid arthritis	6 (2.9)
Walking aide	9 (4.4)

We calculated the FRAX risk score using the US Hispanic, Spain, and Mexican databases. Conformity between the databases was determined by comparing the number of subjects that meet the established threshold for high risk. In the US, NOF guidelines suggest persons with low bone mass and a FRAX score above 3% risk of hip fracture or 20% risk of osteoporotic fracture are at high risk and should be considered for treatment [1]. The conformity of threshold limits was also compared to the risk assessment tool developed by Pluijm et al. [4]. The Pluijm algorithm uses age and the number of risk factors to estimate risk.

## Results

Risk factor data from post-menopausal women in Guatemala City was captured and the fracture risk was assessed by for hip and any type of fracture was calculated using the US Hispanic, Spain, and Mexico calculators. For each database, the mean risk values for hip and for major osteoporotic fracture at any location within 10 years are presented in Table 2. The average 10-year risk for major osteoporotic fracture in Spain was statistically different from US Hispanic and Mexico, as determined by a Wilcoxon signed rank test. The risk was similar for the US Hispanic and Mexico database. The mean risk values for hip fracture were similar between Spain and Mexico, but statistically lower using the US Hispanic database.

**Table 2:** Ten-year fracture risk in Guatemalan women using different FRAX databases.

Any fracture risk: US Hispanic	6.6±6.2%
Any fracture risk: Mexico	6.5±6.5%
Any fracture risk: Spain	5.8±6.5%
Hip fracture risk: US Hispanic	1.8±3.4%
Hip fracture risk: Mexico	2.2±3.6%
Hip fracture risk: Spain	2.2±4.3%

We used the US NOF guidelines for treatment which suggest treatment for patients with a 10-year hip fracture probability ≥3% or a 10-year major osteoporotic fracture risk ≥20%. The number of patients meeting the suggested threshold limits for treatment using the Spain and Mexico calculators were identical. There was 100% conformity in threshold limits for both hip and major osteoporotic fracture risk.

Out of 204 subjects, the FRAX US Hispanic database identified 41 patients at risk of fracture by the NOF guidelines. All 41 individuals were identified as high risk for hip fracture, with 9 of those subjects also meeting the threshold for being at risk of any major osteoporotic fracture. The FRAX Spain/Mexico databases identified 44 individuals meeting the threshold limit. All 44 subjects met the threshold for risk of hip fracture, with 8 of those also meeting criteria for risk of any osteoporotic fracture. Using either the US Hispanic, Spain, or Mexico database, there were no instances in which a subject met the threshold of a 20% 10-yeaar risk of major osteoporotic fracture without also meeting the 3% threshold for risk of hip fracture.

**Figure 1:**
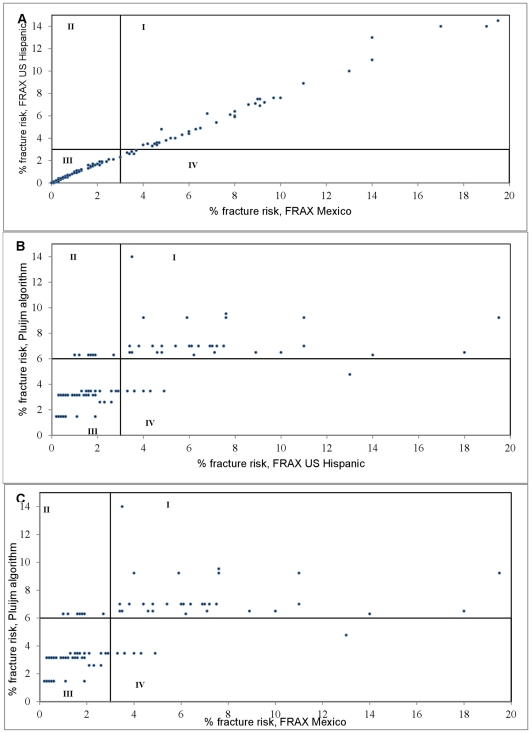
Conformity of risk fracture algorithms for any osteoporotic fracture. FRAX scores normalized to 3% risk threshold of hip. Pluijm algorithm scores are non-continuous and normalized to 6% risk threshold. Quadrant I and III represent conformity at high risk and conformity at low risk, respectively. Quadrant II represents high risk by x-axis algorithm, low risk by y-axis algorithm. Quadrant IV represents high risk by y-axis algorithm, low risk by x-axis algorithm. A) Comparison of risk between US Hispanic and Mexico FRAX database. B) Comparison of US Hispanic FRAX and Pluijm algorithm. C) Comparison of Mexico FRAX and Pluijm algorithm.

In comparing the US Hispanic database to the Spain/Mexico databases, 160 subjects were deemed at low risk by both methods and 41 subjects at high risk by both methods. Three subjects met the threshold according to the Spain/Mexico databases, but not the US Hispanic database. The mean conformity for any fracture risk between US Hispanic and the other two databases was 98.5% (Table 3, Figure 1A). For the risk of any fracture, 80% women (n = 163) do not meet the threshold and 20% (n = 41) of women would be considered for treatment based on the FRAX US Hispanic database. Assessment by the FRAX Spain/Mexico databases show 22% (n = 44) of women meeting the threshold and 78% (n = 160) below it. Comparison by chi-square test denotes that the difference between subgroups' headcount is not statistically significant (χ^2^ = 0.13; p>0.05). Conformity was 99.0% (χ^2^ = 0.66; p>0.05) based on major osteoporotic fracture and 97.5% (χ^2^ = 0.13; p>0.05) based on risk of hip fracture.

**Table 3:** Conformity of risk assessments between FRAX US Hispanic database, FRAX Spain/Mexico database, and Pluijm tool.

Assessment Tool		Number of subjects	conformity at high fracture risk	conformity at low fracture risk	high fracture risk by A and low by B	high fracture risk by B and low by A	Conformity (%)
A	B						
FRAX US	FRAX Spain/Mexico	204	41	160	0	3	98.5
FRAX US	Pluijm	102	31	58	7	6	87.2
FRAX Spain/Mexico	Pluijm	104	32	53	5	12	83.3

In the Netherlands, Pluijm et al developed a simple fracture risk score assessment using risk factors that include age, prior fragility fracture since age 50, body weight <60 kg, use of a walking aid, and smoking. The Pluijm tool separates women into three separate age groups (60–69, 70–79, and 80+) and bases risk on the number of the other risk factors present (Table 2). The Pluijm risk score, similar to FRAX, calculates both the ten-year absolute risk of hip fracture and major osteoporotic fracture. It is important to note that the percent risk deemed high risk for Pluijm does not correspond to the threshold limit of the FRAX calculations.

Of the 102 respondents of age>60, 37 met the criteria for classification of high risk of fracture. Using this subgroup, the US Hispanic database identified 41 subjects at high risk, and the Spain/Mexico databases identified 44 at risk for fracture. In comparing the US Hispanic database to the Pluijm algorithm, 58 subjects were deemed at low risk by both methods and 31 subjects at high risk by both methods. Seven subjects met the threshold according to the US Hispanic database, but not by Pluijm. Six subjects were deemed low-risk by the US Hispanic database but high risk by Pluijm. For the Spain/Mexico and Pluijm comparison, 53 subjects were deemed at low risk by both methods and 32 subjects at high risk by both methods. Five subjects met the threshold according to the FRAX databases, but not by Pluijm. Twelve subjects were deemed low-risk by the Spain/Mexico databases but high risk by Pluijm. The Pluijm evaluation shows conformity of 87.2% (χ^2^ = 0.33; p>0.05) and 83.3% (χ^2^ = 1.0; p>0.05), respectively, when compared to the US Hispanic and Spain/Mexico FRAX results for thresholds for risk of fracture (Table 3, Figure 1A & 1B).

## Discussion

The WHO denotes the US as “very high risk” and Spain as “moderate risk” for the epidemiology of osteoporosis. Mexico is not mentioned. As a reference, Kanis et al. reports that life-time risk of hip fracture is 15.8% in USA, 12% in Spain, and 8.5% in Mexico [5]. Recent studies suggest that the risk of fracture in Guatemala is elevated [3].The risk profiles for the individual countries may be different and yield significantly different results for 10-year risks of hip or osteoporotic fracture, but the conformity of threshold limits suggest that the difference in profiles is inconsequential.

FRAX is a complex algorithm that requires either internet access or numerous paper charts (at least 8 per country). Even in the US, physician access to the internet is limited in patient exam rooms [6]; a deficiency that can be amplified in Guatemala. The assessment tool developed by Pluijm only requires age and the number of risk factors present. This algorithm is simple and shows conformity to the precision of the FRAX calculations.

Although the different FRAX databases provide variations in the absolute risk of fracture, the overall conformity to treatment thresholds amongst the US Hispanic, Spain, and Mexico databases show the database used would have little effect as to the decision to treat. The Pluijm tool conforms to the FRAX thresholds as well. It does not matter which country-specific calculator or assessment tool is used, as there are a similar number of patients that would meet the intervention threshold. Although the FRAX database is more robust and more commonly used, the ability to use the Pluijm algorithm to garner similar results can be useful in developing nations like Guatemala where computer and internet access limits access to more powerful tools.
